# Correction: Sarcopenic obesity in predialysis chronic kidney disease: Muscle strength is a predictor of mortality and disease progression in a six-year prospective cohort

**DOI:** 10.1371/journal.pone.0332435

**Published:** 2025-09-12

**Authors:** The PLOS One Staff

## Notice of Republication

This article was republished on 28^th^ August to correct an error in the article title. The word “mortality” was misspelled.

The correct title is: Sarcopenic obesity in predialysis chronic kidney disease: Muscle strength is a predictor of mortality and disease progression in a six-year prospective cohort.

The correct citation is: Oliveira D, de Souza VA, Souza GC, Suassuna LF, Bastos MG, Reboredo MM, et al. (2025) Sarcopenic obesity in predialysis chronic kidney disease: Muscle strength is a predictor of mortality and disease progression in a six-year prospective cohort. PLoS ONE 20(3): e0318773. https://doi.org/10.1371/journal.pone.0318773

The publisher apologises for the error. Please download this article again to view the correct version. The originally published, uncorrected article and the republished, corrected article are provided here for reference.

## Supporting information

S1 File Originally published, uncorrected article. (PDF)

S2 File Republished, corrected article. (PDF)
