# Supplementary material for: Sarcopenic obesity in predialysis chronic kidney disease: Muscle strength is a predictor of mortality and disease progression in a six-year prospective cohort
Source: PLoS One. 2025 Mar 18;20(3):e0318773. doi: 10.1371/journal.pone.0318773 (PMC11918380; doi:10.1371/journal.pone.0318773)
Supplement: Attachment — Submitted filename: Response_to_Reviewers_auresp_2.docx [file pone.0318773.s003.docx]

Dear Reviewer,

We thank you again for your careful reading, which will improve the article. We have detailed our responses below.

**Comments to the Author**

1. If the authors have adequately addressed your comments raised in a previous round of review and you feel that this manuscript is now acceptable for publication, you may indicate that here to bypass the “Comments to the Author” section, enter your conflict of interest statement in the “Confidential to Editor” section, and submit your "Accept" recommendation.

Reviewer #1: (No Response)

2. Is the manuscript technically sound, and do the data support the conclusions?

The manuscript must describe a technically sound piece of scientific research with data that supports the conclusions. Experiments must have been conducted rigorously, with appropriate controls, replication, and sample sizes. The conclusions must be drawn appropriately based on the data presented.

Reviewer #1: Yes

Thank you for the review.

3. Has the statistical analysis been performed appropriately and rigorously?

Reviewer #1: Yes

Thank you for the review.

4. Have the authors made all data underlying the findings in their manuscript fully available?

The [PLOS Data policy](http://track.editorialmanager.com/CL0/http:%2F%2Fwww.plosone.org%2Fstatic%2Fpolicies.action%23sharing/1/010f0193d2454536-db7876e3-ef48-43a0-938d-bacfb00e49a6-000000/gSfxmUbjMH1qEKXFSF74V-6JTFjqqGTVRKPe6FOsXEw=189) requires authors to make all data underlying the findings described in their manuscript fully available without restriction, with rare exception (please refer to the Data Availability Statement in the manuscript PDF file). The data should be provided as part of the manuscript or its supporting information, or deposited to a public repository. For example, in addition to summary statistics, the data points behind means, medians and variance measures should be available. If there are restrictions on publicly sharing data—e.g. participant privacy or use of data from a third party—those must be specified.

Reviewer #1: Yes

Thank you for the review.

5. Is the manuscript presented in an intelligible fashion and written in standard English?

PLOS ONE does not copyedit accepted manuscripts, so the language in submitted articles must be clear, correct, and unambiguous. Any typographical or grammatical errors should be corrected at revision, so please note any specific errors here.

Reviewer #1: Yes

Thank you for the review.

6. Review Comments to the Author

Please use the space provided to explain your answers to the questions above. You may also include additional comments for the author, including concerns about dual publication, research ethics, or publication ethics. (Please upload your review as an attachment if it exceeds 20,000 characters)

Reviewer #1: A well done project examining the effects of Sarcopenic obesity on a number of variables. Of note it appears that the heading for Non SO and Sarcopenic Obesity are switched for this table 3. This is evident when one looks at the significant measurements near the bottom of the table. For a discussion of table 6. The authors should discuss other papers where moderate to low bmi is associated with increased risk or rate of death. Additionally, providing evidence or explanation as to why grip strength predicts time to death is warranted. Things such as physical activity, nutrition, and rest or sleep!

1- Regarding Table 3, we did not observe the error pointed out by your review. We have provided more details on the title of the table:

Table 3 – Demographic and clinical characteristics of the sample, dichotomized by Sarcopenic Obesity and Non-Sarcopenic Obesity (SO) and we emphasize that this population with sarcopenic obesity has greater fat mass, the data on lean mass refer specifically to sarcopenia.

| **Fat Percentage, % (mean ± SD)**  **Total Body Fat, kg (mean ± SD) Total**  **Abdominal Circumference (cm)** | 43.7(8.1)  34.1(9.5)  101.5(9.3) | 35.1(8.6)  23.4(7.9)  90.2(10.1) | <0.0001  <0.0001  <0.0001 |
| --- | --- | --- | --- |

2- Regarding Table 6, we have added another paragraph on low BMI and mortality in this population. This data has been discussed in the literature for some time and reference 28 already mentions it. We have added the following studies:

When we approach pre-dialysis patients, there is a lack of studies in the literature, and a study by Kovesdy et al showed that lower BMI is associated with greater mortality in patients with CKD not yet on dialysis therapy. Adjustment for case-mix and surrogate markers of malnutrition and inflammation attenuated, but did not reverse, this relationship. This study was carried out on a sample of 521 men followed for a period of 5.5 years. In contrast, Madero et al evaluated 1759 individuals in relation to BMI and progression and mortality in CKD and found no association. These studies demonstrate that there is no consensus on this variable and reevaluating it in other contexts is of utmost importance.

Kovesdy CP, Anderson JE, Kalantar-Zadeh K. Paradoxical association between body mass index and mortality in men with CKD not yet on dialysis. Am J Kidney Dis. 2007 May;49(5):581-91. doi: 10.1053/j.ajkd.2007.02.277. PMID: 17472839

Madero M, Sarnak MJ, Wang X, Sceppa CC, Greene T, Beck GJ, Kusek JW, Collins AJ, Levey AS, Menon V. Body mass index and mortality in CKD. Am J Kidney Dis. 2007 Sep;50(3):404-11. doi: 10.1053/j.ajkd.2007.06.004. PMID: 17720519.

Regarding the handgrip, the first study we found (Chang YT) evaluated 128 clinically stable patients with non-dialytic CKD and followed up for 33.8 ± 9.2 months demonstrating that handgrip is an independent predictor of composite renal outcomes in non-dialytic CKD patients. This study suggest that handgrip can be incorporated into clinical practice for assessing nutritional status and renal prognosis in patients with non-dialytic CKD. Handgrip strength is suggested as an indirect assessment of nutritional status in CKD patients, but evidence is limited for non-dialysis-dependent CKD patients. As cross-sectional study included 404 patients from the Phase II KoreaN Cohort Study for Outcome in Patients With CKD. Higher handgrip was significantly associated with lower malnutrition risk after adjustment (per 1 standard deviation increase, adjusted odds ratio, 0.47 [0.30–0.75]) (33x).(KIM et al)

Chang YT, Wu HL, Guo HR, Cheng YY, Tseng CC, Wang MC, Lin CY, Sung JM. Handgrip strength is an independent predictor of renal outcomes in patients with chronic kidney diseases. Nephrol Dial Transplant. 2011 Nov;26(11):3588-95. doi: 10.1093/ndt/gfr013. Epub 2011 Mar 28. PMID: 21444362.

Kim M, Park YW, Im DW, Jeong Y, Noh HJ, Yang SJ, Kang E, Ryu H, Kim J, Koo JR, Na KR, Seong EY, Oh KH. Association of Handgrip Strength and Nutritional Status in Non-Dialysis-Dependent Chronic Kidney Disease Patients: Results from the KNOW-CKD Study. Nutrients. 2024 Jul 26;16(15):2442. doi: 10.3390/nu16152442. PMID: 39125323; PMCID: PMC11314453.

About non-pharmacological interventions to improve physical function in adults with end-stage renal disease, one systematic review and network meta-analysis for randomized controlled trials of a total 44 eligible trials enrolled 2,250 participants, and 16 interventions were identified. It was found that combined resistance and aerobic exercise is the most effective intervention with improvement in several parameters including handgrip and fatigue (Zhao et al).

Zhao Q, He Y, Wu N, Wang L, Dai J, Wang J, Ma J. Non-Pharmacological Interventions to Improve Physical Function in Patients with End-Stage Renal Disease: A Network Meta-Analysis. Am J Nephrol. 2023;54(1-2):35-41. doi: 10.1159/000530219. Epub 2023 Mar 30. PMID: 36996785.
