# Supplementary material for: Sarcopenic obesity in predialysis chronic kidney disease: Muscle strength is a predictor of mortality and disease progression in a six-year prospective cohort
Source: PLoS One. 2025 Mar 18;20(3):e0318773. doi: 10.1371/journal.pone.0318773 (PMC11918380; doi:10.1371/journal.pone.0318773)
Supplement: Attachment — Submitted filename: Response to Reviewers.docx [file pone.0318773.s002.docx]

Reviewers' comments:

Reviewer's Responses to Questions

**Comments to the Author**

1. Is the manuscript technically sound, and does the data support the conclusions?

The manuscript must describe a technically sound piece of scientific research with data that supports the conclusions. Experiments must have been conducted rigorously, with appropriate controls, replication, and sample sizes. The conclusions must be drawn appropriately based on the data presented.

Reviewer #1: Yes

Reviewer #2: Partly

2. Has the statistical analysis been performed appropriately and rigorously?

Reviewer #1: Yes

Reviewer #2: Yes

3. Have the authors made all data underlying the findings in their manuscript fully available?

The [PLOS Data policy](http://c05y1x9s.r.us-east-2.awstrack.me/L0/http:%2F%2Fwww.plosone.org%2Fstatic%2Fpolicies.action%23sharing/1/010f01922b36ec9d-8958052f-7cc6-4bb1-9283-e734bfa175dd-000000/O4BYK_GhOUAZTkPMZjMI_sUtFyQ=178) requires authors to make all data underlying the findings described in their manuscript fully available without restriction, with rare exception (please refer to the Data Availability Statement in the manuscript PDF file). The data should be provided as part of the manuscript or its supporting information, or deposited to a public repository. For example, in addition to summary statistics, the data points behind means, medians and variance measures should be available. If there are restrictions on publicly sharing data—e.g. participant privacy or use of data from a third party—those must be specified.

Reviewer #1: Yes

Reviewer #2: Yes

4. Is the manuscript presented in an intelligible fashion and written in standard English?

PLOS ONE does not copyedit accepted manuscripts, so the language in submitted articles must be clear, correct, and unambiguous. Any typographical or grammatical errors should be corrected at revision, so please note any specific errors here.

Reviewer #1: Yes

Reviewer #2: Yes

**5. Review Comments to the Author

Please use the space provided to explain your answers to the questions above. You may also include additional comments for the author, including concerns about dual publication, research ethics, or publication ethics. (Please upload your review as an attachment if it exceeds 20,000 characters)**

**Reviewer #1: Excellent manuscript and fine publication on this complicated topic.**

Dear Reviewer,

We are grateful for the thorough and attentive review, and we have made the pertinent changes for the purpose of improving our manuscript.

The authors,

**Abstract
Include low and moderate BMI and death results.**

We appreciate the suggestion and we have added these results to the abstract.

Results: Sixteen percent of our sample were diagnosed with SO, but this was not associated with death or RRT, while lower BMI values were negatively associated with these outcomes. However, in an isolated analysis, handgrip strength was correlated with both RRT (p=0.029) and death (p=0.003). We recommend that health professionals pay greater attention to muscle strength, striving for earlier assessment, in an effort to better counsel patients and implement actions to increase or preserve strength through nonpharmacological treatment, such as resistance training.

**Methods
Define kg/f**
This is a relevant remark, the kilogram-force is a widely utilized unit of measurement in studies assessing hand grip strength. The kilogram-force is defined as the magnitude of the force exerted by Earth's gravity (at sea level on latitude 45º) on one kilogram of mass, which is approximated to 9.8 Newton of force.

We added a definition to the text:

Handgrip strength was used to assess muscle strength using a Jamar Hydraulic Hand Dynamometer® with a scale ranging from 0 to 90 kg and a resolution of 2 kg. Low muscle strength was determined by values less than 30 kg-f (Kg-f is the measure of force applied by human movement to a dynamometer) and 20 kg-f for men and women, respectively.

**Discussion
The results from table 6 ie. BMI 18.5-24.9 and BMI < 18.5 should be discussed. These data suggest that low and moderate BMI is a cause for concern as is low grip strength.**

This is a useful remark. We found no association between handgrip strength and BMI analyzed as a continuous or categorical variable (r=0.032; p=0.700). We have added this to the manuscript:

A recent review [27] has shown that SO is associated with a higher incidence of DM, incapacity and mortality in certain populations. We did not observe an association between SO and DM in our study; however, the same was not true for the outcome of death, with obesity being a protective factor. This finding corroborates the findings of the Brazilian Peritoneal Dialysis Multicentric Cohort Study (BRAZPD) [28], which also showed a greater risk of mortality in patients with a lower BMI (< 18.5 kg/m^2^). Among CKD patients, higher BMI values (normal weight and overweight) have been shown to be protective against negative outcomes and patients in nutritional risk are in need of rigorous assistance [28]. eGFR was protective against RRT outcome, as expected.

**Reviewer #2: This study aims to assess the associations of predialysis CKD patients with sarcopenic obesity and its components with death and disease progression to renal replacement therapy. This is an important topic and the information gained has the potential to positively benefit both patients with CKD and clinicians.

However, there are several concerns that need to be addressed by the authors.**

Dear reviewer,

Your rigorous review has greatly contributed to the improvement of the manuscript. We have taken all remarks into consideration and hope to have satisfactorily answered all of them.

The authors

**Lines 58-59: reference is needed.**

To corroborate the remark, we have referenced an article that was already utilized for the manuscript:

**Obesity is a multifactorial chronic disease with a complex etiology and is directly associated with increased morbidity and mortality (5)**. This affirmative is present in the fifth reference.

**Lines 69-71: “The main findings regarding SO in patients with CKD are a direct relationship with albuminuria, even in patients with normal BMI”. I was not able to locate the reference in which SO was directly related to CKD patients with normal BMI. Please include. Also, by definition, individuals with a normal BMI are not obese. Therefore, is it possible to have sarcopenic obesity if you have normal BMI?**

Reference 15 corroborates with this remark: “These findings suggest that decreased muscle mass and coexistence with excess adiposity show associations with CKD and high eGFR even in adults with normal BMI. Body composition measured by DXA could provide information on the relationship of body composition with CKD and high eGFR.” **(**Hyun YY, Lee KB, Rhee EJ, Park CY, Chang Y, Ryu S. Chronic kidney disease and high eGFR according to body composition phenotype in adults with normal BMI. Nutr Metab Cardiovasc Dis. 2016 Dec;26(12):1088-1095. doi: 10.1016/j.numecd.2016.09.003. Epub 2016 Sep 17. PMID: 27776918.)

Regarding sarcopenic obesity (SO) in patients with normal BMI, it is a possible occurrence, because BMI is incapable of discriminating between muscle mass and fat mass. This means that diagnosing obesity only through the BMI is inherently imprecise. In our study, our methodology for defining SO is present in line 123, in which we describe the use of three indexes: BMI, FMI and BFP. **“**When assessing SO, the FNIH criteria were utilized in combination with the three aforementioned variables (BMI, FMI and BFP). Based on these data points, the sample was dichotomized into SO and non-SO groups.”

**Ensure all abbreviations used consistently throughout the manuscript and are defined at first use. For example, FNIH and EWGSOP are not define within the text.**

We are grateful for the remark and we have added definitions to the text.

In accordance with the Foundation for the National Institute of Health (FNIH) criteria...

Table 1- European Working Group for Sarcopenia in Older People (EWGSOP)., However, we observed that this definition is not utilized in the text and removed it from the manuscript.

**Line 104: decimal places should be consistente**

It has been corrected.

**Methods:
No rationale is provided for why both the EWGSOP and FNIH criteria were used. Also, only the FNIH criteria was used to assess SO and for analysis. Please provide clarification as to why EWGSOP was included in the methodology or why it was not used to assess SO and included in the analysis.**

Both criteria were utilized for phase 1: In recent years, different definitions of sarcopenia have been proposed, and there is no consensus on its characterization. The diagnostic criteria of the European Working Group on Sarcopenia in Older People (EWGSOP) were proposed in 2010 and are widely used in clinical practice and research. However, new definitions for sarcopenia have recently been proposed by the Foundation for the National Institutes of Health (FNIH) Sarcopenia Project . (Souza VA, Oliveira D, Barbosa SR, Corrêa JODA, Colugnati FAB, Mansur HN, Fernandes NMDS, Bastos MG. Sarcopenia in patients with chronic kidney disease not yet on dialysis: Analysis of the prevalence and associated factors. PLoS One. 2017 Apr 27;12(4):e0176230. doi: 10.1371/journal.pone.0176230. PMID: 28448584; PMCID: PMC5407780.). The EWGSOP criteria were revised after phase 1, so we removed its definition and opted to utilize the FNIH criteria, which has not been modified since.

**Participants were recruited from 2014-2022. The first phase of the study consisted of a cross-sectional evaluation of patients carried out between 2014-2016 which also included 100 participants (lines 90-93). Therefore, it seems no additional participant were recruited from 2017-2022, is this accurate? Please also include how many participants were excluded during the enrollment period and the reasons why such participants did not meet inclusion criteria.**

The first phase inclusion criteria for study enrollment included being an ambulatory male or female adult who were aged over 18, with CKD on conservative treatment. Contraindications to participate in the study included patients with limited performance of tests necessary for assessment of muscle strength and function due to severe neuropathy, liver disease, stroke sequelae, arthritis, arthrosis, amputations, severe chronic obstructive pulmonary disease, Parkinson’s disease, cancer, and HIV/AIDS.” (CKD was d Souza VA, Oliveira D, Barbosa SR, Corrêa JODA, Colugnati FAB, Mansur HN, Fernandes NMDS, Bastos MG. Sarcopenia in patients with chronic kidney disease not yet on dialysis: Analysis of the prevalence and associated factors. PLoS One. 2017 Apr 27;12(4):e0176230. doi: 10.1371/journal.pone.0176230. PMID: 28448584; PMCID: PMC5407780.).

We added to the manuscript:

The first phase of the study consisted of a cross-sectional evaluation of the patients carried out between 2014 and 2016 and was published as the original article *“Sarcopenia in patients with chronic kidney disease not yet on dialysis: Analysis of the prevalence and associated factors”* [4]. In the second phase of our study, the same patients from the first phase were enrolled and there was no loss of follow up.

**Lines 123-125: “FNIH criteria were used in combination with BMI, FMI, and BFP to assess SO”. It is not clear how SO was diagnosed. What specific variables were used? And if multiple body composition variables were used as indicated, how did this change the prevalence of SO based on the variable used? Also, is there precedent for the method used in this study? If so, please include reference.**

Once again your remarks have helped to improve the manuscript. We have added the definitions to the paragraph.

When assessing SO, the FNIH criteria were utilized in combination with the three aforementioned variables (BMI, FMI and BFP). Based on these data points, the sample was dichotomized into SO and non-SO groups. Regarding body fat, in case BMI and FMI or BFP reached discordant values, FMI and BFP measurements were prioritized (19,20).

**Please expand upon methods for how grip strength and gait speed were measured. For example, was grip strength determined in the dominant limb or both limbs. Were participants in the seated or standing position? Where participants instructed to walk at their customary gait speed?**

We are grateful for the remark. We more clearly defined our methodology for assessing these variables.

Handgrip strength was used to assess muscle strength using a Jamar Hydraulic Hand Dynamometer® with a scale ranging from 0 to 90 kg and a resolution of 2 kg. Three measurements were taken from the dominant limb, while the patient was sitting on a chair, with the elbow flexed in 90º, with a three minute interval between each measurement, the highest measurement was utilized for our analysis. Low muscle strength was determined by values less than 30 kg-f (Kg-f is the measure of force applied by human movement to a dynamometer) and 20 kg/f for men and women, respectively. In accordance with the FNIH criteria, low muscle strength was considered for values below 26 kg-f or 16 kg-f for men and women, respectively. To assess muscle performance, a 6-meter gait speed test was utilized, and values less than 0.8 m/s were considered poor performance. The gait test was performed in an ample and well lit corridor, a measuring tape determined the length of the track plus 2 meters before the starting point and 2 meters after the finish, in order to discount the patient's acceleration and deceleration. Individuals were instructed to walk at their usual pace, and time was measured by research staff.

**Statistical analysis:
Lines 151-153: “associations were analyzed with Pearson correlation coefficient.” This information does not seem to be presented in the manuscript.**

We performed several statistical tests that are not present in the manuscript. Reviewer #1 suggested that we should add one of these analyses in the manuscript, so we opted to keep the statistical analysis paragraph as is and add the results suggested by Reviewer #1.

**Results:**
**Lines 162-163: “Obesity was present in 36%, 28% of the sample had sarcopenia, and 16% had SO”. Please consider editing this sentence for clarity.**

We modified the manuscript : Out of the total of the sample, 28% had sarcopenia, 36% were diagnosed with obesity and 16% had SO.

**Table 4: authors indicate triglycerides to be significantly different between groups at a p-value of 0.054. This does not meet the level of significance as proposed in the statistical analyses section (p-value < 0.05).**

We modified the manuscript.

... profile in SO patients, with higher glucose levels (Table 4).

**Figure 1 quality makes it difficult to read and interpret.**

We have sent another file with higher quality.

**Its unclear what added information Tables 5 and 6, please consider removing.**

In nephrology, there is an important epidemiological discussion regarding what is usually called "reverse epidemiology" among dialysis patients, for whom a higher BMI is protective against mortality. This is scrutinized by some as a survivorship bias that could be explained by overweight pre-dialysis patients having higher mortality, and that those who reach the dialysis stage have some other protective factor. However, in this research, we had the opportunity to assess mortality in pre-dialysis patients and observed that, even before renal replacement therapy, a higher BMI was protective against mortality. This weakens the idea of survivorship bias and reinforces the idea of a "reverse epidemiology" among dialysis patients. It is important to demonstrate that these results are not associated solely to BMI, thus we believe that it is important to keep tables 5 and 6. Reference 28 of the manuscript: **(**28. Fernandes NM da S, Bastos MG, Franco MRG, Chaoubah A, Lima M da G, Divino-Filho JC, et al. Body size and longitudinal body weight changes do not increase mortality in incident peritoneal dialysis patients of the Brazilian peritoneal dialysis multicenter study. Clinics. 2013;68(1):51–8.)

**Lines 197-199: please include abdominal circumference values in Table 1 if measured.
Were differences in body composition and FNIH outcomes assess between SO and non-SO groups? This information is not presented in the Results.**

We agree that this information is relevant, however table 1 includes only the FNIH criteria for sarcopenia. Therefore, this information was added to table 2.

Abdominal circumference (cm): 92.07 (10.05)

Regarding the differences in body composition between SO and non-SO patients, by definition muscle mass is reduced in the SO group. This information has been added to table 3.

SO	non-SO

| **Fat Percentage, % (mean ± SD)**  **Total Body Fat, kg (mean ± SD) Total**  **Abdominal circumference (cm)** | 43.7(8.1)  34.1(9.5)  101.5(9.3) | 35.1(8.6) p<0.0001  23.4(7.9) p<0.0001  90.2(10.1) p<0.0001 |
| --- | --- | --- |

**Discussion:
Lines 211-212: “Higher levels of IL-6 were found in the SO group than non-SO group”. This statement does not seem to be true given that there were no significant differs between groups in IL-6 according to Table 4 or lines 168-170. Please revise.**

Inflammatory marker levels were not statistically significant and we emphasized it in the results. We have added the same emphasis on the discussion:

While not statistically significant, higher levels of IL-6, were found in the SO group than in the non-SO group, which might associate inflammation with the development of SO. The mechanism underlying sarcopenia is unclear, and several theories suggest that, in addition to older age, other factors, such as a reduction in anabolic hormones, an increase in myofibril apoptotic activity, oxidative stress, and an accumulation of proinflammatory cytokines, might play a role [25]. IL-4 has been shown to be relevant for preserving muscle mass and improving insulin sensitivity in mice [26]; however, its effects on muscle tissue energy metabolism remain unclear. The present study showed that, even if not significantly, the SO group had higher values of IL-4, more investigation is needed to better comprehend this marker's role in sarcopenia (26).

**Lines 212-216: Have the authors given consideration to the differences between primary (aging) versus secondary (disease-induced) sarcopenia and how this might influence the interpretation of the findings?**

Our sample's average age was high (73.59 years) and every participant was previously diagnosed with Chronic Kidney Disease (CKD), therefore it is hard to make a robust inference into how age and CKD were independently associated with sarcopenia. What leads us to believe that CKD played a greater role in sarcopenia are the multivariate analysis results, especially after adjusting for age and glomerular filtration rate (GFR), in which GFR was shown to be more important for some of the outcomes. However, this conclusion is not robust, because patients without CKD were not included in the analysis. Thus, this conclusion is more an opinion than a proper inference, and we chose not to include it in the manuscript.

**Lines 218-220: again, the data do not seem to support this statement. Please revise.**

We agree that the data was not statistically significant, as was stated in the text. We have modified the paragraph:

The present study showed that, even if not significantly, the SO group had higher values of IL-4, more investigation is needed to better comprehend this marker's role in sarcopenia (26).

**Lines 224-226: “greater risk of mortality in patients with lower BMI”. This statement deserves greater attention and discussion regarding the obesity paradox seems warranted.**

We agree with your observation and have added it to the text.

A recent review [27] has shown that SO is associated with a higher incidence of DM, incapacity and mortality in certain populations. We did not observe an association between SO and DM in our study; however, the same was not true for the outcome of death, with obesity being a protective factor. This finding corroborates the findings of the Brazilian Peritoneal Dialysis Multicentric Cohort Study (BRAZPD) [28], which also showed a greater risk of mortality in patients with a lower BMI (< 18.5 kg/m^2^). Among CKD patients, higher BMI values (normal weight and overweight) have been shown to be protective against negative outcomes and patients in nutritional risk are in need of rigorous assistance [28]. eGFR was protective against RRT outcome, as expected.

**Lines 234-244: this paragraph seems to lump together myosteatosis and strength. Although there is the potential for some interaction between these concepts, they are distinct from one another.**

We agree with this remark, and we have made it clear that we are hypothesizing about a possible contribution to reducing hand grip strength.

Myosteatosis is a recent concept that is defined by the infiltration of fat into skeletal muscle tissue. A possible mechanism for the association between obesity and myosteatosis is that adipocytes may become saturated, leading to an increase in ectopic fat storage. In muscle tissue, these ectopic fat storages release proinflammatory cytokines, resulting in local inflammation. This adipose muscular infiltration occurs in great proximity to muscle fibers; therefore, myosteatosis and obesity may amplify each other, leading to greater loss of muscle function, this may contribute to reducing hand grip strength [31].

**What are the clinical implications of the findings of this work? Given the importance of strength, what interventions should be considered? Has resistance exercise been shown to beneficial for preserving muscle health and strength in CKD patients?**

We appreciate the commentary and these considerations are present in the conclusion of the manuscript.

**Conclusion**

In our study, there was a SO prevalence of 16% in the predialysis CKD population, but it was not associated with RRT or death outcomes. When analyzing the various components of sarcopenia, it was possible to observe a correlation between handgrip strength and both outcomes. These findings show the importance of early assessment of muscle strength in predialysis CKD patients. This approach would allow for the implementation of actions aiming to preserve or increase muscle strength utilizing nonpharmacological strategies, such as resistance training.

6. PLOS authors have the option to publish the peer review history of their article ([what does this mean?](https://c05y1x9s.r.us-east-2.awstrack.me/L0/https:%2F%2Fjournals.plos.org%2Fplosone%2Fs%2Feditorial-and-peer-review-process%23loc-peer-review-history/1/010f01922b36ec9d-8958052f-7cc6-4bb1-9283-e734bfa175dd-000000/7xP6nPBybt4YobGbvxH7yPjQFOs=178)). If published, this will include your full peer review and any attached files.


If you choose “no”, your identity will remain anonymous but your review may still be made public.


**Do you want your identity to be public for this peer review?** For information about this choice, including consent withdrawal, please see our [Privacy Policy](https://c05y1x9s.r.us-east-2.awstrack.me/L0/https:%2F%2Fwww.plos.org%2Fprivacy-policy/1/010f01922b36ec9d-8958052f-7cc6-4bb1-9283-e734bfa175dd-000000/A06qycUg6RgMO81Kv-Y4wZr3QGs=178).

Reviewer #1: **Yes:** Charles Paul Lambert, PhD

Reviewer #2: **Yes:** Jared M. Gollie

[NOTE: If reviewer comments were submitted as an attachment file, they will be attached to this email and accessible via the submission site. Please log into your account, locate the manuscript record, and check for the action link "View Attachments". If this link does not appear, there are no attachment files.]

While revising your submission, please upload your figure files to the Preflight Analysis and Conversion Engine (PACE) digital diagnostic tool, [https://pacev2.apexcovantage.com/](https://c05y1x9s.r.us-east-2.awstrack.me/L0/https:%2F%2Fpacev2.apexcovantage.com%2F/1/010f01922b36ec9d-8958052f-7cc6-4bb1-9283-e734bfa175dd-000000/-9vzLuGgzdzEhE-ATrewsElV2bE=178). PACE helps ensure that figures meet PLOS requirements. To use PACE, you must first register as a user. Registration is free. Then, login and navigate to the UPLOAD tab, where you will find detailed instructions on how to use the tool. If you encounter any issues or have any questions when using PACE, please email PLOS at [figures@plos.org](mailto:figures@plos.org). Please note that Supporting Information files do not need this step.
